# Would the combination of everolimus with endocrine-therapy help in FGFR2 positive serous endometrial cancer?

**DOI:** 10.18632/oncoscience.168

**Published:** 2015-06-05

**Authors:** Maria Rosa Cappelletti, Letizia Gnetti, Daniele Santini, Daniele Spada, Stephen B. Fox, Daniele Generali

**Affiliations:** ^1^ U.S. Terapia Molecolare e Farmacogenomica, AO Azienda Istituti Ospitalieri di Cremona, Cremona, Italy; ^2^ Department of Onco-Haematological and Internal Medicine, Section of Pathology University of Parma, Parma, Italy; ^3^ University Campus Bio-Medico Roma, Oncologia Medica, Roma, Italy; ^4^ Department of Anatomical Pathology, Peter MacCallum Cancer Centre, East Melbourne, Victoria, Australia

**Keywords:** FGFR2, endometrial cancer, everolimus, FGFR inhibitors

## INTRODUCTION

Chemotherapy is a critical approach in the treatment of advanced or recurrent endometrial cancer. The use of chemotherapy is now the standard treatment for women with early-stage disease. However, there are no agents approved by the US Food and Drug Administration for the metastatic setting of endometrial cancer, which has a very low chance to be treated with success. Due to the great expectation on novel targeting agents in advanced endometrial cancer, new drugs against specific molecular pathways with particular focus on PI3KCA/AKT/mTOR axis are emerging as promising treatments for endometrial cancer with aggressive phenotype [1]. The very issue is the lack of progress in cancer treatment due to the heterogeneity and the genetic complexity of many tumours leading to an urgent knowledge of the molecular profile of an individual's tumour to guide appropriate treatment selection.

We have read with enthusiasm the article by Slomovitz et al published in Journal of Clinical Oncology on January 26, 2015 where it was reported the increased clinical benefit rate with the combination of letrozole and everolimus in women with recurrent endometrial cancer along with a manageable toxicity [2]. Moreover, patients with endometriod histotype carrying the CTNNB1 mutations underwent to a good response to treatment. On the counterpart the serous histology did not respond with the same rate to mTOR inhibition; moreover they have not identified based on the molecular analysis they performed a possible responsive-subtype of the serous cancer. The Cancer Genome Atlas has led to the molecular reclassification of endometrial cancer. These genomic advances are determinant for the proper selection of the adequate therapy in the future of the patient care. In the genomic era, clinicians and molecular oncologists should invest their efforts in finding “druggable” targets for the available drugs on the market. In our clinical experience, letrozole alone is already able to modulate the mTOR axis in breast cancer [3] suggesting its use also in other endocrine-related cancer such as estrogen receptors positive endometrial cancer. In our experience, the molecular analysis of the primary tumor or of the metastasis from endometrial cancer showed low percentage rate of mutations of PIK3CA and KRAS according on what reported by Slomovitz et al [2].

**Figure 1:**
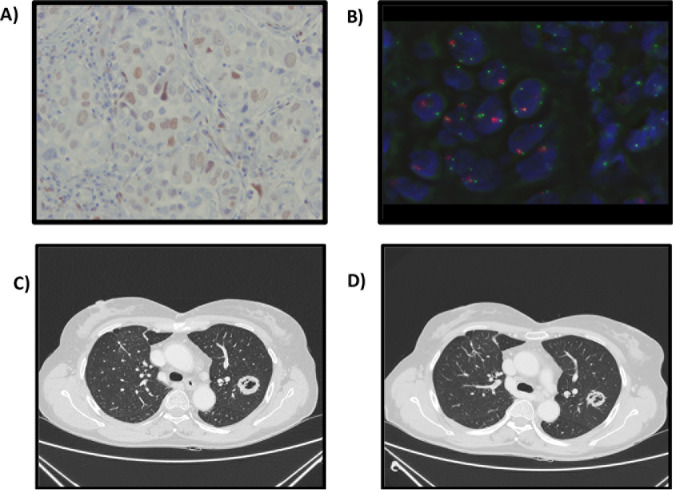
Biological and radiological features: A) Estrogen Receptor, B) FGFR2 Amplification; C) Target lesion at basal CT scan, D) Target lesion after 12 months of treatment

As previously reported that from the minimal genetic variations in the PI3K or FGFR pathways or CCND1, derives the most benefit from EVE based-therapy (HR = 0.27 vs 0.40 for the full NGS population) [4], in three serous endometrial cancers not responsive to conventional therapies, we applied for further molecular analysis, a part KRAS, PIK3CA and proliferation related genes, with particular regards to the staining of the estrogen receptor and FGFR2 FISH-based evaluation (Figure 1 A e B). The combination of 1gr medroxyprogesterone acetate daily and 10 mg everolimus daily induced stable radiological response of the liver metastases and partial radiological response in lung metastases during the time course of treatment in 2 patients with FGFR2 amplification (Figure 1 C and D). No response was detected in 1 patient without amplification of FGFR signal. Amplification or mutation of fibroblast growth factor receptor (FGFR) has been reported in endometrial cancers with a frequency of 10% and FGFR2 may represent a novel molecular target for the treatment of endometrial cancer [5]. Activation of FGFR induces PI3K and AKT activities through recruitment and tyrosine phosphorylation of the docking protein Gab1 that results in the activation of PI3K [6]. In our FGFR2+ve patients, we obtained, accordingly to the mRECIST Criteria, stable disease longer than 12 months in line with the positive data from the recent progression free survival analysis from the BOLERO-2 trial [7]. These results suggest that longer stable disease maybe achieved in the absence of a direct alteration in the PI3K/AKT/mTOR pathway, even when multiple molecular aberrations are present. However, FGFR2 may modulate the PIK3CA/AKT/mTOR axis perhaps explaining in part the response. Slomovitz et reported that only few patients with serous endometrial cancer responded to the combination of letrozole/everolimus and maybe they were FGFR 1 or 2 amplified.

Thus, the combination of everolimus and aromatase inhibitors has the change to show some activity in the particular subtype of EC as the FGFR2+ve serous carcinoma. Tumors with ER expression are dependent upon downstream growth factor signaling and may respond better with the addition of molecular inhibitors based on the proper target selection. Indeed, our reports encourage the perspective investigation of combination of everolimus along with hormone-therapy in pre-treated FGFR2+ recurrent endometrial cancer patients. Specific molecular analysis with focus on FGFR signalling in serous endometrial cancer is mandatory.
